# On the Epidemic Ague or "Fainting Fever" of Persia, a Species of Cholera, Occurring in Teheran in the Autumn of the Year 1842

**Published:** 1843-10

**Authors:** Charles W. Bell

**Affiliations:** Physician to Her Majesty's Mission to Persia


### REPORT on the EPIDEMIC AGUE or "FAINTING FEVER" of PERSIA, *A Species of Cholera, occurring in Teheran in the Autumn of the year* 1842.

By Charles W. Bell, M.D.
Physician to Her Majesty's Mission to Persia.*

The epidemic which has been of late so fatally prevalent in several districts of Persia, particularly in the capital Teheran, and in the neighbouring district of Cazveen, presents some points of analogy with Indian cholera (cholera asphyxia), both in its nature and habits. It seems to be following the same north-westerly course which that disease pursued when, some years ago, it made its first appearance in Europe: and it may be regarded as a direct continuation of that form of cholera which was prevalent at the mouths of the Indus, in the commencement of the year 1842; and which reached Bunder Abbass, in the Persian Gulf, a little later in the season. I have been assured by the prime minister of the King of Persia, who is the best authority to be had here on the subject, that a disease similar to that which I am about to describe, has appeared at several points in succession between Bunder Abbass and Teheran; and it was evidently making its progress west and northerly in the direction of Azerbijan, when it was checked, by the setting in of winter, on the high grounds that intervene.

If, unfortunately, this disease should extend itself farther, some previous knowledge of the nature of the complaint cannot fail to be interesting to practitioners; for though not perhaps very formidable when the principles upon which it is to be treated are understood, and sufficient time is given to carry them into practice, still it is far from contemptible. According to the best calculation I can make, it would appear that not less than four fifths of the whole population of this city were attacked by it, and that one sixteenth (of the whole population) have died of it.† It is perhaps true that the mortality would have been much less if the native physicians had adopted a proper line of practice; still a disease producing such havoc, under any circumstances, is a formidable one. And if by publishing the present account of it, I should be fortunately able to save to others the regret which I myself have experienced for the loss of my first patients attacked with it before I became acquainted with its nature (for subsequent experience has convinced me that these ought to have been saved), it will make some amends for the anxiety which I then underwent; for, without previous knowledge of its nature, in a disease so obscure at its commencement as this is, and with a termination so unlooked for, some lives must be sacrificed, even in the best hands, before the necessary experience be gained.

### I. History of the Disease.

The disease of which I am about to give some account is, in my opinion, essentially a quotidian ague; but, as far as I know, it has not hitherto been described as an epidemic.‡ Although dangerous when left to nature, it is most amenable to correct treatment. It becomes more dangerous in proportion as the ordinary stages, shivering, fever, and sweating, are less distinctly marked. And, when in place of the shivering fit, there occurs an agonizing oppression of the heart with general congestion of the venous system, the case will, according to my experience, always prove fatal if unassisted by art; whereas in the very worst cases, unless the circulation have become absolutely stagnant, this congestion may be relieved and by proper means a recurrence of it be prevented.

The following are the principal modes in which this disease produces its dangerous or fatal effects: first, general venous congestion, and, consequently, impeded action

* This Report was drawn up at the request of Count Medem, H. I. M. the Emperor of Russia's Minister Plenipotentiary at the Court of Teheran.

† The population of Teheran ranges in different months between 700,000 and 100,000 inhabitants.

‡ I should here except an allusion to what is apparently the same disease mentioned as occurring in a district of India, in 1825, in Mr. G. H. Bell's work on Cholera, p. 22. It is to his instruction that I am indebted for the rationale of bleeding in cholera.

of the heart and cessation of the circulation ; second, sudden effusion into the lungs and exudation from the capillaries generally into the cellular tissue ; third, diseased spleen, constantly recurring ague, general dropsy, and failure of the powers of life.

The other sources of danger, which no doubt exist, I have been prevented from ascertaining by the impossibility of making any post-mortem examination in this country. But I have little doubt that one of the most common causes of sudden death was the effect of venous congestion upon a heart enfeebled by previous disease.

In describing this disease I shall divide it into several varieties, without pretending that there are not others, or that all I shall notice are always distinct and distinguishable from one another. I shall take the varieties as nearly as possible according to the number of persons so affected, beginning with the simplest and most ordinary form.

*First variety.* In this variety the disease assumed the form of a simple quotidian ague, and affected more than four fifths of the whole population of Teheran. This is a form of ague which I have rarely seen in Persia, the usual type being tertian. In general the patient got well in two or three days without medicine ; but if he dwelt in a low or damp situation, or if he were treated with the usual Persian prescription of manna dissolved in two or three quarts of the juice of water melon, or if he indulged in fruit, the disease usually passed into the second degree or variety.

*Second variety.* This was also quotidian ague but accompanied with considerable pain on pressure over the spleen and very rapid enlargement of that organ ; attended with remarkable blanching of the lips and a pale, clean, bloodless tongue. The rigor was shorter and less severe than usual, but the fever continued long and, though not running high, was accompanied with much thirst and dryness of the tongue. The sweating stage was short and imperfect, and the intermission of short duration. In some cases this fever was attended with severe headach, and was then liable to pass into the third degree ; or the shivering ceased and vomiting and purging came on instead of the cold fit, or it assumed some other severer form ; or if recovery took place, constantly recurring fever, always accompanied by pain on the left side, at every change and full of the moon, or on the slightest error of diet, were too often the consequences of it. I daily, however, met with cases where this fever has continued, with little intermission, for two or three months ; the powers of life gradually sinking under the disease : dropsy or dysentery being the usual termination.

*Third variety.* This form had several modes of commencement. Sometimes it began at once, by the patient becoming suddenly insensible without previous symptoms ; at other times, it was preceded by formal ague. In many instances, again, the patient would suffer for some time previously from intermitting headach daily increasing, and great want of sleep ; he would then have one attack of ague, and, next day, at the same time, would sink down insensible. This was the form of disease from which the greatest number of deaths took place, and obtained for the malady its Persian name TAB-I-GHASH, or "fainting fever." During the insensibility the pulse was feeble and the extremities cold. From this state many were never roused; but if they were, the pulse gradually attained power and the patient came slowly to his senses, complaining of intense headach and a feeling of oppression at the heart; a low kind of fever then came on which was succeeded by very imperfect perspiration, generally confined to the head and chest. Next day, about the same hour, insensibility returned, and each attack continuing longer than the preceding one, the period of death depended upon the strength of the patient or violence of the disease ; most frequently, however, death took place on the third attack. As the end approached the secretion of urine ceased,* the efforts of the heart at reaction became feebler, the skin felt like that of a corpse, cold and damp, the body became purple and mottled, and the pulse became less perceptible at the wrist : at length the patient was seized with tetanic convulsions and died. In these cases, as often observed in cholera, the feet began to get

* On one occasion I was called to see what was said to be a case of retention of urine, and found the patient in articulo mortis from this disease, the body cold and the legs beginning to get warm. I attempted to bleed, but only ℥iij could be obtained by drops. He was soon seized with convulsions, and died.

warm shortly before death, and just as the warmth had spread up the legs and reached the trunk the patient died. Indeed, were other symptoms wanting, I should consider warmth commencing in the feet while the rest of the body was cold, quite sufficient to mark the case as hopeless.

*Fourth variety.* Owing to their brief duration, I saw few cases of the severer form of this variety; but I received the details of many such cases as the following: the person was seized with sudden pain in the pit of the stomach, the belly at the same time becoming hard; there was neither vomiting nor purging, but the patient craved incessantly for ice, and died in about an hour.

The cases presenting such symptoms as the following, were probably milder attacks of the same kind. In this form there was no insensibility, no shivering, little or no perceptible fever, and no perspiration; the primary characteristic symptoms were a fixed pain in the pit of the stomach, extreme tenderness on pressure over the left lobe of the liver and region of the spleen, and extreme tension of the abdominal muscles. One or both of the recti abdominis became hard as a board, continuing for days in a state of constant tension, but without any painful cramps or spasms. Nearly at the same hour each day the patient was observed to become exceedingly anxious and restless, tossing from side to side, sighing and throwing the arms above the head, as in yawning, and the pulse became very small and frequent, and the body damp and cold. By and by, this oppression passed off, the body resumed its natural warmth, and the pulse nearly its natural volume, but this continued quicker than usual, and then to all appearance the patient had very little the matter with him. Each day, however, the oppression of the circulation became greater and the attack continued longer; the pulse now became weaker and an ice-cold exudation ran off the brow and back of the hands.* The struggling of the heart to overcome the load of blood which oppressed it was most painful to listen to,—now almost overcoming the obstruction, the pulse for an instant gaining power, and a partial warmth spreading over the surface; and, again, the force of the heart succumbing to the disease, and the icy coldness—much colder than death—returning. The craving for iced water was incessant so long as this state lasted. The evacuations meantime were bilious, and the quantity of urine daily diminished, and at length ceased altogether. At length the intermission between the attacks of oppression ceased to occur, the pulse was only perceptible at intervals, and the patient, who up to this time had been perfectly sensible and even able to walk to stool, fell into a state of stupor. The skin now became blue and mottled, and the patient gradually sunk or died in convulsions. Here also, as I remarked above, sometime before death took place the lower limbs recovered almost their natural warmth. In all the cases I saw of this variety there was much feeling of distension of the stomach and inactivity of the bowels, and sometimes a little vomiting.

*Fifth variety.* Here the disease was cholera, with the usual symptoms of vomiting and purging, and suppression of urine. The two former symptoms, however, were less in degree than in the ordinary Indian cholera; and the painful cramps of the limbs were either absent or less in degree. For about ten days cases of vomiting and purging of an intermittent kind were very frequent; as if a slight attack of cholera had taken the place of the cold fit of ague: generally, however, these latter cases yielded easily to the chalybeate draught with quinine, to be hereafter mentioned.

*Sixth variety.* I, myself, saw only one well marked case of this singular form of the disease, although I heard of several occurring in Teheran. This form was said to be especially fatal in a regiment quartered in the district of Boorojird; but perhaps I may be mistaken in classing it with this disease on premises so slight. According to the account which I received, the patients went to bed in good health, felt chilly in the

* It was my painful duty to attend an intimate friend, the Shah's chief physician, through an illness of this kind. He was one of the first attacked, and had not yet seen a case of the disease. He obstinately refused to submit to bleeding or to take the medicines which I recommended, having a great predilection for calomel. It is chiefly from his case that I describe the latter stages of this complaint. All that I subsequently attended were cured.

night, and next morning their head and neck were found swollen to an enormous size, they breathed with difficulty, and in the course of a few hours died suffocated.

The case which I saw was that of a courier just arrived from Boorojird. He was apparently quite well at night, and was to have started with dispatches in the morning, when, not making his appearance, he was sent for, and was found with his face and neck immensely swelled and almost suffocated. He was brought to me immediately, when I found that, besides the extraordinary anasarca of the head and neck, which was rapidly extending upon the chest, there was effusion going on into the lungs. The skin was cool, the pulse about ninety, soft and weak, and the tongue rather pale. I ordered an immediate large bleeding; blood was obtained with the greatest difficulty at first, but presently flowed freely, and he felt much relieved. He then took calomel and jalap in syrup, which was swallowed little by little with some difficulty. These operating smartly, there were no remains of the edema left next morning. Being quite recovered, he started on the following day and had no recurrence of the attack. The symptoms here were too obscure to enable me to say whether this ought to be considered as the cold stage of the disease, but the difficulty of obtaining blood inclines me to this belief.

Besides the above more formal varieties of this epidemic, there were observed several other forms less distinctly marked, or the same forms characterized by the predominance of particular symptoms or combinations of symptoms. Under this head I may enumerate—intermittent pain and swelling over the tibiæ; tic douloureux and nervous pains of almost every part of the body; tonic spasms, continuing for many days, of a portion of the colon, with obstinate constipation, but without other severe symptoms; jaundice, &c. &c.* What confirmed the accuracy of the view which classed these latter affections with the general epidemic, was the fact that they, in general, yielded easily to the same medicine which was found so effectual in it.

### II. Treatment of the Disease.

As this Report is intended to be strictly of a practical character, it forms no part of my present object to enter, at any length, either upon the etiology or pathology of the disease of which I have given an account. Before proceeding, however, to notice the plan of treatment which I found most successful, I shall make one or two remarks which seem to have a direct and important bearing on the subject.

In the first place I have to state that while the disease was at its height in the town, the blood, even of those not sensibly attacked, was universally of a dark, dusky, reddish-brown colour, very different from that of healthy venous blood, and in general the serum did not separate from the clot. This fact there was abundant opportunity of verifying, for most Persians are in the habit of losing blood twice or oftener in the year, and a great number let blood once or twice every month. Secondly, in those affected with the severer forms of the disease, the blood drawn in the cold fit was always grumous, coming at first slowly or in drops, and coagulating as soon as drawn, even at the mouth of the wound; and no separation of the serum took place. Thirdly, during the epidemic the urine of the people in general was much darker coloured than at other times, while in those who were seriously affected it was, if secreted at all, like porter, and in very small quantity.

From these facts, as well as from the general history of the epidemic given above, it would appear that in the production of this disease there must have been some occult cause acting upon the blood, and producing effects similar, on the whole, to those which we find in cholera. The most marked of these effects are, 1, impaired fluidity and sluggishness of the blood in the veins; 2, consequent probably on this state of the blood, great general venous congestion; 3, a disposition to a daily periodical return

* A kind of jaundice was common during the height of the epidemic, which, in the absence of other symptoms, I attributed to spasms of the gall-duct, and, as there were slight symptoms of ague, treated with the chalybeate draught alone, to which it yielded easily. In the fever that I saw in the island of Karrach in 1839, all died jaundiced: dissection showed nothing in the liver, but in every one the spleen was soft, and broken with the slightest touch.

of this congestion; 4, in most, perhaps in all cases, more or less of disease of the spleen. In many instances pain is felt in the region of the spleen before the occurrence of any other symptom.

If these views are at all correct, or whatever be the mode of explaining them, if the four states there named be of constant occurrence in this disease, the attention of the practitioner must ever be directed to the means of obviating them, and he must not allow himself to be distracted from their consideration by other collateral symptoms of less importance.

In the treatment of this disease there were several obvious indications the means of fulfilling which seem to me to be these:

1. In the first place, it is essential to avoid everything likely to increase the disease. Under this head, among the most powerful are damp, and the use of fruit and uncooked vegetables, especially water-melons. The last in many cases I have seen acting as a direct poison, bringing on an immediate attack of fever, which terminated fatally in two or three hours. Therefore, removal to an upper room and careful diet I found to be particularly useful.

2. For the purpose of obviating the tendency to venous congestion, iron appears to me to excel every other medicine. The sulphate is the preparation to which I have given the preference, on account of its solubility and the ease with which it combines with sulphate of magnesia or aloes—one of which is necessary, both on account of the astringent quality of the iron, and because I found the bowels in this complaint generally either inactive or exceedingly irregular in their action.*

3. With a view to obviate the periodical attacks, quinine is obviously the medicine most to be depended upon. Alone, however, and uncombined, quinine appeared to be too exciting, increasing the thirst, headach, and pain at the pit of the stomach; and it produced astonishingly little specific effect upon the disease.

What I found by far the most effectual mode was to combine both these tonics with a cooling laxative, and give them all at the same time. For this purpose I made use of the following formula with little alteration in nearly a thousand cases, and with the most complete success:

℞ Magnesiæ sulphatis ℥j

Quinæ sulphatis gr. ix

Ferri sulph. gr. xij

Acid sulph. dilut. ʒj

Aquæ fontis Oiss. Dosis ℥ij.

I directed a dose of this to be taken, in ordinary cases, three or four times a day,—if possible at the fourth, third, second, and first hour before the daily accession; but if this took place either in the night or early in the morning, or if the time of the attack were uncertain, I directed the medicine to be taken at stated intervals during the day, whatever might happen to be the stage of the fever at the time. So far from increasing the thirst and fever, as quinine uncombined often does when taken in the hot stage, the effect of this combination was generally immediate in subduing the thirst and dryness of the mouth, which were always distressing. Thus administered, the mixture almost always produced its effect in cutting short the fever on the third day, and this, too, in cases where large doses of quinine alone had failed.

In the severer cases I reduced the quantity of sulphate of magnesia, and sometimes gave the medicine every half hour.† Very commonly the effect of the first few doses was to *increase* the strength and duration of the *shivering fit*, and *shorten the fever* of the first paroxysm; it then prevented the accession of the next fit: and comparatively few had a third attack after commencing its exhibition. I found it of great

* The value of iron in subduing irregular action of the circulation in general, or of an organ, as in uterine complaints, where it is alike useful in amenorrhea and in menorrhagia, has been often remarked. For its efficacy in the cure of spleen disease, I have only to refer to the testimony of Mr. Twining's invaluable work on the Diseases of Bengal.

† On two or three occasions I was surprised to find my patients, especially one who was very dangerously ill, recover much more quickly than I expected; but it afterwards came out that they had finished the whole supply of medicine at a single draught.

use, where pain in the region of the spleen was considerable, to aid its effect by the application of six or eight leeches, a few hours before the cold stage was expected: in cases of young children leeches were particularly beneficial.

In a great many cases, when the patients lived in a low or damp situation, the recurrence of fever was so frequent, especially just before the full and change of the moon,—each attack being accompanied with more and more enlargement and pain in the region of the spleen,—that I was obliged in several houses to lay it down as a general rule for those who had suffered, that on the 12th or 13th, and on the 26th or 27th of the Persian or lunar month, six or eight leeches should be applied to the region of the spleen, or six or seven ounces of blood taken from the arm, and the use of chalybeate medicine resumed for two or three days. I here allude especially to one district of the town where scarcely any one escaped fever, and particularly to several houses of one street under which a watercourse of sulphurous-smelling water ran, and where every one attacked, who was not either treated in this manner or escaped to another quarter of the town, died. Many of the survivors, notwithstanding this advice, which was too disagreeable to be literally followed after the immediate danger was over, are still suffering from fever at every change and full of the moon.

But cases occurred where no time was given for the comparatively slow operation of this medicine. This was where the attack was sudden and severe, or where the oppression, which unless relieved immediately is sure to be fatal, had become so great before assistance was sought, that it was quite beyond the power of medicine. Here there is plainly no resource but the lancet. But in order to use this with success, both the fullest confidence in the correctness of our views of the pathology of the disease, and some boldness too are necessary. It must be confessed that appearances are much against the practice; and our resolution is very apt to fail us when we see the man cold, with scarcely a pulse; his lips blanched and bloodless; himself anxious and terrified, and tossing his arms like one who is already bleeding to death. Very naturally, too, his friends object to a proposal so little according with their prejudices. If in this state a vein be opened freely, the blood comes only in drops or trickles in a feeble stream, dark and grumous or of a brownish colour, and coagulating on the arm. When about two or three ounces were drawn in several such desperate cases I found the patients get faint and throw themselves about in an agony of oppression; the blood stopped and they appeared dying in my hands; and I have no doubt that they would have died had the attempt here been abandoned. In such cases, strong ammonia must be held to the nostrils, the arms must be kneaded, and the patient must in some way be induced to cough or sneeze, whereby the blood receiving an impulse again begins to trickle, each moment acquiring force, till at length it flows in a full stream. The patient now sits quietly instead of tossing about in an agony as before; his faintness goes off, and, as the blood flows, he feels his spirits rise, all his oppression ceasing, and his conviction is now complete that he is cured. And so he generally is, if only enough of blood has been taken; but less than sixteen or twenty ounces will not be sufficient. I generally continue the use of the medicine for some days after the bleeding, especially if near the full of the moon,* when relapse is exceedingly likely to occur. But I have seen some very bad cases cured without any further after-treatment than a dose or two of castor oil.

Prejudiced, as I confess I had been, against the practice of bleeding in the cold stage of intermittent fever, having never till this season met with a case which really

* I am well aware of the ridicule to which I expose myself in thus attributing such marked effects on disease to the changes of the moon; but the evidence before me is so strong as to have removed all my doubts upon the subject; and I see the effects in such a number of patients who suffer from ague every full and change, that I should feel ashamed did the fear of ridicule prevent me stating a fact of which I am so entirely convinced, and which, if true, is so important. Unfortunately, no account is kept of the daily number of deaths in Teheran; if there were, I should be content to rest the settlement of the question upon that alone. The 15th I am satisfied has been by far the most fatal day of the lunar months corresponding to September, October, November, and December 1842, and the 12th and 13th, and the 26th and 27th, the days upon which a recurrence of fever has most frequently taken place, or upon which the symptoms have most frequently become aggravated.

required it, I at first only used the lancet in the worst and most hopeless cases of this disease. When, however, my stock of quinine began to run low, I was under the necessity of finding a substitute for it; and the lancet was that which most naturally suggested itself. But being particularly desirous to avoid interfering with the prejudice entertained by the people of this country against bleeding in the cold stage, I determined to try whether the same effect would not result from taking blood *before* the accession of the cold fit. I reasoned thus:—It was more probable, I thought, that the shivering itself was only a symptom marking a certain degree of venous congestion, than that the venous congestion and the shivering commenced at the same moment, and that congestion had probably existed for a considerable time, gradually increasing before it attained the degree necessary to produce so violent a proof of its existence; that if this were the case, I did not see any reason why bleeding should not put a stop to congestion before it had attained what may be called *the shivering point*, and while yet the circulation had power, quite as effectually—or even more so—as it would relieve the congestion when it had attained its maximum; and when, consequently, the circulation was most oppressed and enfeebled. Besides, the quantity of blood stated by the best authorities as necessary to be taken when bleeding in the cold stage is practised, and which my own experience had fully confirmed, was greater than what is usually ordered to be taken at once in Persia, and more than I was often willing to take from my patients during the prevalence of an epidemic of this character; and I was in hopes that a smaller quantity of blood taken at an earlier period of congestion would produce the same effects as a full bleeding in the cold stage. Being careful, then, to ascertain as exactly as possible the usual hour of accession, two hours before the attack was expected I took twelve ounces of blood and gave three grains of quinine immediately afterwards, and I had the satisfaction to find that the blood was easily obtained, without fainting or annoyance, and that no attack followed. I afterwards found that it was of little consequence at what period blood was taken, provided the sweating stage were fairly over; only that when too near the time at which the shivering should commence, it sometimes *hastened* the attack; but, then, it seldom recurred. I was usually in the habit of giving a little quinine or a cup of ginger-tea after the bleeding.

The advantages of this treatment were—that the disease was generally less likely to recur than when cured with medicine alone; and the blood was obtained without difficulty, without any disagreeable faintness, and without alarm to the patient.

In the intermittent or scarcely-intermittent headaches so prevalent at one time, bleeding sometimes cured them at once; and sometimes it was followed soon afterwards by a regular paroxysm, which did not however in general recur. This difference of effect was probably owing to the time at which the blood was taken with reference to the cold stage (which I supposed to exist in these cases, although nothing of the sort was evident to the senses,) there being nothing to show whether the accession of headache belonged to the cold or feverish state of ague.

In the severer cases the period of accession was sometimes so obscure that it was scarcely possible to discover it. The attack of oppression continued so long and went off so gradually,—there was so little fever, and altogether so little of the character of ague in them,—that the patient himself was not aware of the periodical character of the disease. I have generally found it easier to make out from the statements of the patient and his friends the time at which remission took place, than from my own observation directed to the periods of fever. For example: the patient is described as tossing about and extremely restless for eight or ten hours, then, although not apparently better, for some time he is comparatively still, so that he allows the coverlet to remain upon him; perhaps he himself after this perceives a warm moisture on the neck and chest, and then, feeling himself better, enjoys some repose (sleep was very rare); that on the following day about the same time he begins to toss about as before, &c. Here it is evident that the restlessness corresponds with the period of oppression or the cold stage, and that the period of repose is the remission. Having, therefore, begun the exhibition of the medicine immediately, it is the duty of the physician to be present at the time that appears to be the period of remission, when, if he have hit the proper moment, he will find the patient astonishingly well, the skin natural, the pulse quicker and weaker than it should be, but little that is remarkable except pain on pressure over the pit of the stomach; presently, however, the patient's face becomes anxious, he stretches his arms, and frequently throws himself from side to side sighing. If the practitioner have listened to the heart on his first arrival, and does the same now that its struggling has commenced, he will be in no douht as to the correctness of his diagnosis: the period of venous congestion has begun, and now is the moment to use the lancet with success; unless the symptoms be sufficiently mild to permit of his waiting for the remission of the following day, in order to take blood before the oppression comes on. But it is seldom safe to wait; for even if no immediate danger be apprehended, it is to be remembered that this long-continued congestion is inevitably disorganizing the spleen as well as probably the liver and the kidneys, and is very likely to injure the valves of the heart.

I cannot too much urge the importance of most accurately ascertaining the time of the accession (which is more difficult in practice than appears in description), because if blood be taken at the wrong time the effects are most disastrous.

If the intention of bloodletting in this disease be not, as in inflammation, to diminish the force of the circulation, but, on the contrary, by means of abstracting some of that blood which is stifling the right side of the heart and impeding its action, to aid the efforts of nature to overcome this impediment and to give force to the circulation by restoring the balance of action between the right and left side of the heart,—it is quite evident that that object cannot be attained when the obstruction has been overcome and the balance has been restored,—in other words, that an attempt to remove what does not exist is an absurdity, and that whatever harm bloodletting may do after the oppression has been overcome, it can do no possible good.

If the hot stage have commenced it is a proof that instead of the balance of the circulation being on the venous side as formerly, it is now on the arterial side; and although, *theoretically*, bleeding from an artery might now be admissible, bleeding from a vein must necessarily tend still further to disturb the balance,—just as, during the period of congestion of the venous system, opening an artery instead of a vein would obviously still further disturb the balance between the venous and arterial system, which it is our object to restore.* But this fever is the effect of reaction, or, in other words, of that excitation of the circulating organs which is produced by and necessary to overcome the opposition to a free circulation of the blood; and we know that it will presently subside and be followed by a period of weakness, relaxation, and sweating: it is natural, therefore, to suppose that bleeding in either of these stages will tend only to increase the relaxation, to produce fainting, and to increase the tendency to collapse in the subsequent accession. This, I am sorry to say, I know from experience; for, in the first two cases I saw of this disease, not aware that it was an ague and urged by severe local symptoms, I bled in what I now know to be the hot stage: the blood flowed with perfect freedom, but both patients died collapsed soon afterwards.

The history of these and some other cases would be very illustrative of all that I have advanced, as well as of the obscurity of the symptoms; but this report has already drawn to such a length that I must forbear. In terminating it I have chiefly to regret that the prejudices of the country, by rendering post-mortem examinations impossible, have prevented me adding anything to the morbid anatomy of the disease; for many of the cases of dropsy and other of its chronic effects have been very interesting and very obscure.

To conclude, I cannot too strongly recommend the combination of iron with purgatives during the prevalence of this epidemic, whenever the tongue is pale, as especially at its commencement. Until I adopted this practice I found that class of medicines either not acting at all or doing so with undue violence; and that that addition of carbonate or sulphate of iron rendered their operation much more regular and more certain. But it is only when the tongue is pale and clean and bloodless that the good effects of iron are apparent. These effects will be partly evinced by the in-

* Mr. Twining, in his chapter on Cholera, relates two cases which well illustrate this subject,—one, where he bled just as the pulse was rising; the other, where blood was drawn from the radial artery: in both immediate collapse followed.

creased redness of the tongue, sometimes perceptible even in a few hours. If, however, on the other hand, there be a bilious fur upon the tongue, and it and the lips are red and rosy, iron does positive harm by producing excitement. This is especially to be guarded against as the epidemic is passing away, and people in general are returning to their natural state of health.

On the other hand, calomel and all preparations of mercury are especially contra-indicated by the bloodless tongue and pain and swelling of the spleen. Although recommended by some of the highest authorities in Europe, calomel has proved injurious in every case of affection of the spleen, and in all the cases of this disease in which I have tried it; and also, very remarkably, in a remittent fever (in which also the tongue was pale) that was prevalent in the summer months before this epidemic made its appearance.

During the stage of collapse I have seen no benefit from the exhibition of stimulants of any sort. They appeared to me always to do harm, each dose causing increased oppression of the heart and coldness of the extremities.

After recovery the diet should be very light and the quantity of animal food allowed sparing.

It is worthy of remark that during the prevalence of this epidemic, for upwards of three months, I did not meet with a single case of acute inflammation nor of gout or continued fever, although in the same months of the preceding year these diseases, with ophthalmia, enteritis, acute rheumatism, and pericarditis, were the most prevalent complaints.

*Teheran; January,* 1843.

---

